# Cytochrome P450 inhibition potential and initial genotoxic evaluation of 14-O-[(4,6-diaminopyrimidine-2-yl)thioacetyl] mutilin

**DOI:** 10.1038/s41598-020-70400-8

**Published:** 2020-08-10

**Authors:** Yunxing Fu, Yunpeng Yi, Yuan Fan, Ruofeng Shang

**Affiliations:** 1 Key Laboratory of New Animal Drug Project, Gansu Province, Key Laboratory of Veterinary Pharmaceutical Development, Ministry of Agriculture and Rural Affairs, Lanzhou Institute of Husbandry and Pharmaceutical Sciences of CAAS, Qilihe District, No. 335, Lanzhou, 730050 People’s Republic of China; 2 Henan University of Animal Husbandry and Economy, Zhengzhou, 450046 People’s Republic of China

**Keywords:** Drug development, Drug discovery, Molecular medicine

## Abstract

14-O-[(4,6-Diaminopyrimidine-2-yl)thioacetyl] mutilin (DPTM) is a promising drug candidate with excellent antibacterial activity against Gram-positive bacteria. The present study was designed to characterize its Cytochrome P450 (CYP) enzymes inhibition activities and the genotoxicity with the standard Ames test. We determined the inhibitory effects of DPTM on CYP1A2, CYP2D1/6, CYP2E1, CYP2C11/9 and CYP3A/4 in rat liver microsomes (RLMs) and in human liver microsomes (HLMs). The mRNA expressions of the above CYP isoforms and their transcriptional regulators were also evaluated using the Hep G2 cell model. The results showed DPTM exhibited a moderate inhibitory potential against CYP3A/4 (IC_50_ values of 10 ± 2 and 8 ± 2 μM, respectively) and weak against the other CYP enzymes based on their IC_50_ values. Compared to the control, CYP isoforms and their transcriptional regulators mRNA expressions significantly increased when the Hep G2 cells were treated with DPTM for a certain period of time. In the Ames test, *Salmonella strains* TA97, TA98, TA100, TA102 and TA1535 were treated with or without the metabolic activation (S9). Analysis showed the average number of revertant colonies per plate was less in double in the groups treated with DPTM than that in the negative control plate and showed no dose-related increase.

## Introduction

**Figure 1:**
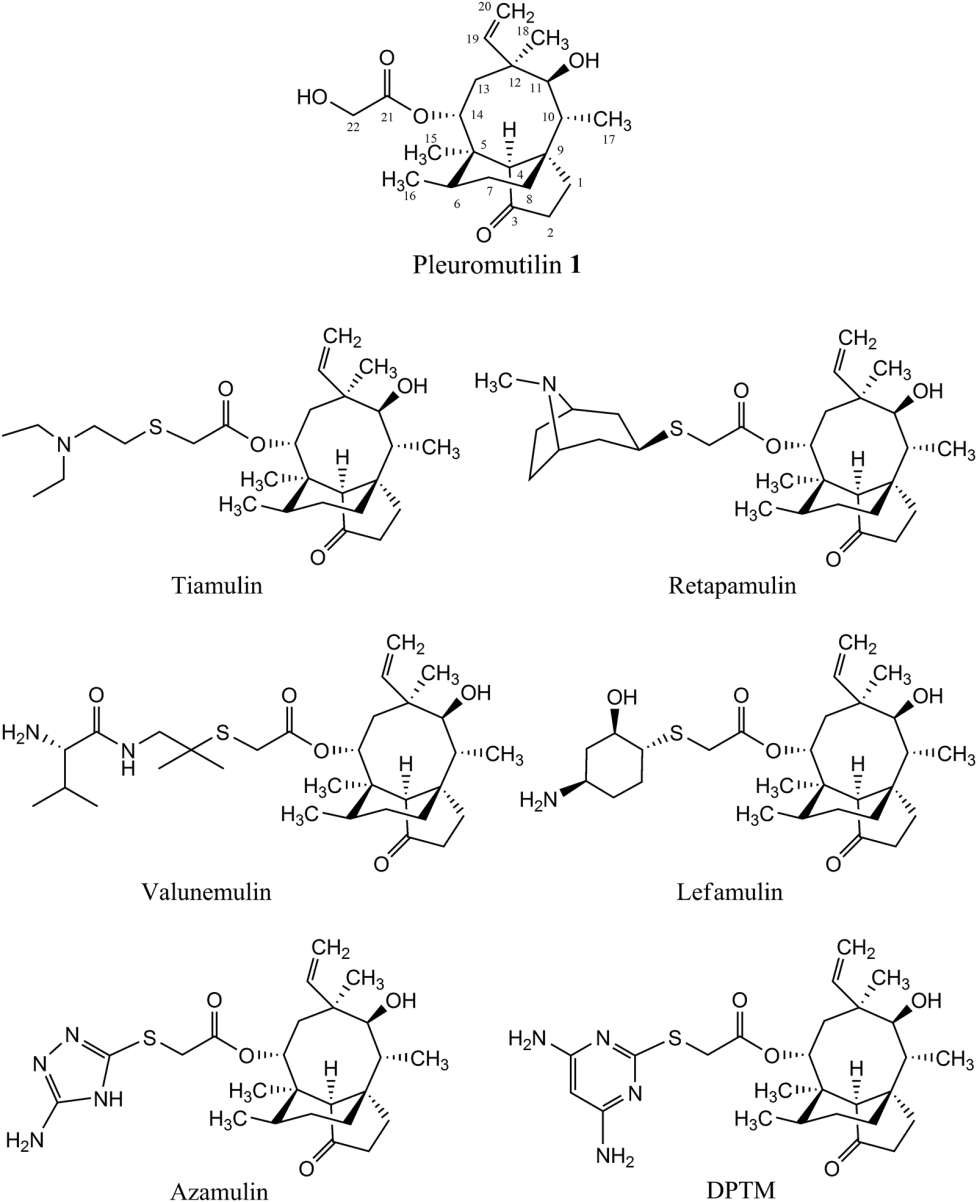
Structures of pleuromutilin, pleuromutilin drugs and 14-O-[(4,6-Diaminopyrimidine-2-yl)thioacetyl] mutilin (DPTM).

Pleuromutilin (Fig. 1), a 5-6-8 tricycle diterpene, was first discovered and isolated from two basidiomycete species in 1951^1^. Pleuromutilin class of antibiotics selectively inhibits bacterial protein synthesis by interacting with the peptidyl transferase centre (PTC) of prokaryotic ribosomes at the A- and P-site through hydrophobic interactions and hydrogen bonds^2–4^. The modifications of pleuromutilin side chain led to tiamulin (Fig. 1) and valnemulin (Fig. 1) for veterinary use^5,6^. Retapamulin (Fig. 1) is a topically administered pleuromutilin that was first approved for human use in 2007^7,8^. Lefamulin (BC-3781, Fig. 1) developed by Nabriva Therapeutics was approved by FDA for human use in 2019^9,10^.

Azamulin (Fig. 1), an azole pleuromutilin derivative designed for human use, has entered phase I clinical trials in volunteers in 1980s^11^. Unfortunately, azamulin did not undergo further clinical trials because of its Cytochrome P450 (CYP) inhibition and limited bioavailability^12,13^. Tiamulin is also a potent inhibitor and inducer of CYP, via the formation of a metabolic intermediate complex^14^. Lefamulin showed relative high inhibition for CYP3A4 with IC_50_ < 5 μM^15^. Therefore, the investigation of inhibition potency of pleuromutilin analogues against CYP was subsequently emphasized to avoid the risk for development a new drug.

A novel pleuromutilin derivative, 14-*O*-[(4,6-Diaminopyrimidine-2-yl)thioacetyl] mutilin (DPTM, Fig. 1), was synthesized and reported in ours previous work^16^. This compound showed the excellent in vitro activities against Gram-positive bacteria, including methicillin-resistant *Staphylococcus aureus* (MRSA), *Staphylococcus aureus* (*S. aureus*), *Bacillus subtilis* (*B. subtilis*), as well as in vivo activity using systemic infection mode in mice^16^. As previous studies reported that pleuromutilin analogues showed high CYP inhibition effect^17,18^, we evaluated the inhibitory effects of DPTM on the major CYP enzymes, including CYP1A2, CYP2D1, CYP2E1, CYP2C11 and CYP3A in rat liver microsomes (RLMs) and CYP1A2, CYP2D6, CYP2E1, CYP2C9 and CYP3A4 in human liver microsomes (HLMs). Furthermore, mRNA expressions of CYP enzymes and their transcriptional regulators, including the aryl hydrocarbon receptor (AhR), the pregnane X receptor (PXR) and the peroxisome proliferator-activated receptor (PPAR) were investigated after treatment by DPTM using the human HepG2 cell-line. Moreover, the genotoxic properties of DPTM were also assessed by the standard Ames test with and without metabolic activation (S9).

## Results

### Cytotoxicity

**Figure 2:**
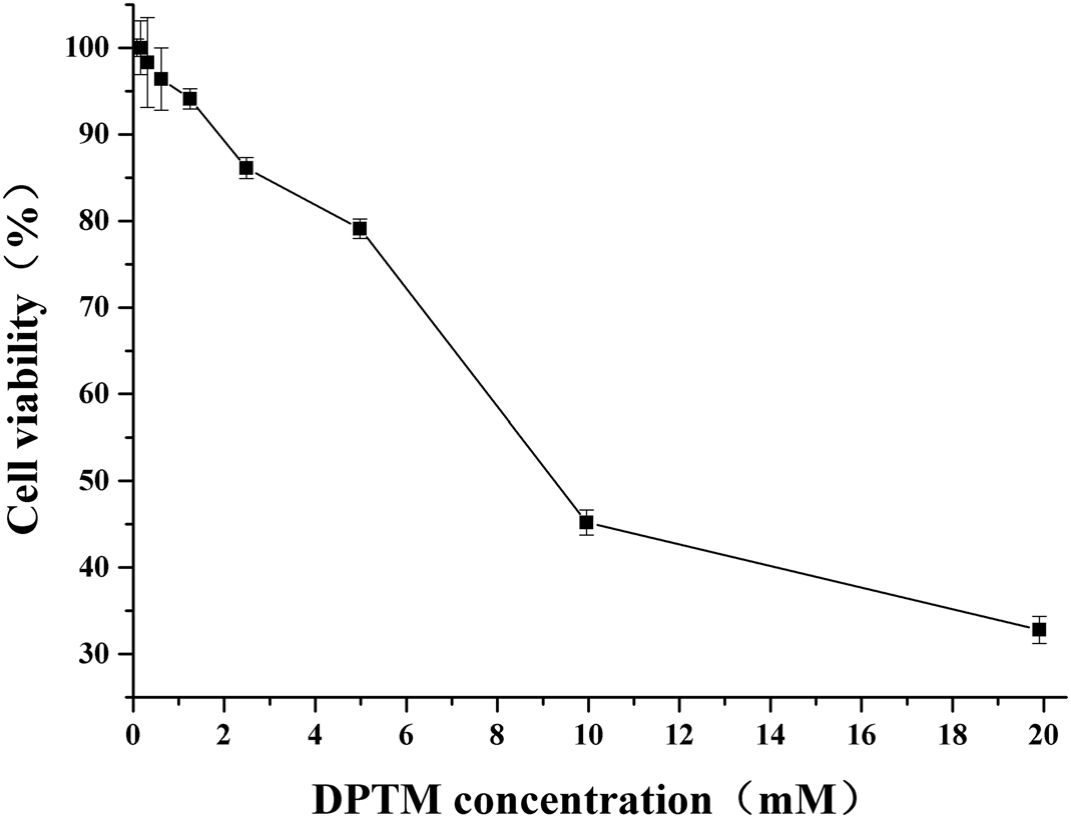
Cytotoxicity of DPTM for Hep G2 cells tested by MTT methodology. Each data point represents the mean value ± SD (n = 3).

Using the MTT (3-(4,5-dimethyl-2-thiazolyl)-2,5-diphenyl-2H-tetrazolium bromide) assay, the cytotoxic potential of DPTM was evaluated on Hep G2 cells. The viability percentages of cells treated with different dilutions of DPTM were shown in Fig. 2. The result was represented as the 50% inhibition (IC_50_) value. On the whole, along with the increase of concentrations of DPTM, the cell viability became lower with the IC_50_ value of 10 ± 2 mM.

### Effect of drug on cytochrome P450

**Figure 3:**
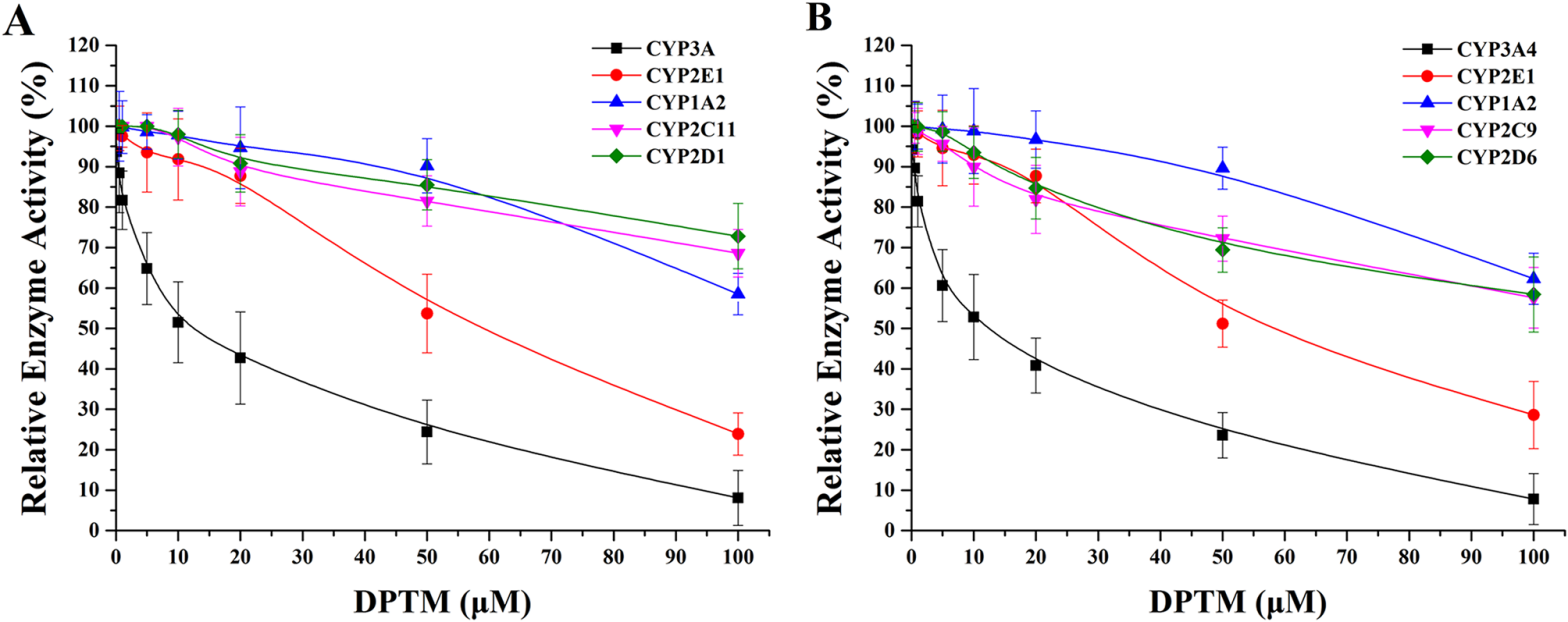
Inhibitory effects of DPTM on CYP isoforms in RLMs (**A**) and in HLMs (**B**). Each data point represents the mean value ± SD (n = 3).

We used cocktail method to assess the direct inhibitory potential of DPTM on CYPs in RLMs and HLMs. The determined IC_50_ values with 95% confidence intervals from eight-point concentration–response curves (Fig. 3) were listed in the Table 1. Ketoconazole and fluvoxamine used as the positive controls for the inhibition of CYP3A4 and CYP1A2, respectively, showed strong activities with IC_50_ values (0.18 and 0.33 μM, respectively) within the reference ranges^19^. In RLMs and HLMs, DPTM exhibited moderate inhibitory potentials against CYP3A and CYP 3A4 with IC_50_ values of 10 ± 2 and 8 ± 2 μM, respectively. In the cases of CYP1A2, CYP2D1/6, CYP2E1 and CYP2C11/9, DPTM showed weak inhibitory effects with IC_50_ ≥ 61 ± 8 μM.

**Table 1:** Inhibitory effects of DPTM on CYP isoforms in RLMs and HLMs.

Isoform in RLMs	IC_50_ values (μM)	Isoform in HLMs	IC_50_ values (μM)
CYP1A2	> 100	CYP1A2	> 100
CYP2D1	> 100	CYP2D6	> 100
CYP2E1	61 ± 8	CYP2E1	67 ± 6
CYP2C11	> 100	CYP2C9	> 100
CYP3A	10 ± 2	CYP3A4	8 ± 2

### Effect of DPTM activation on cytochrome P450 mRNA expression

**Figure 4:**
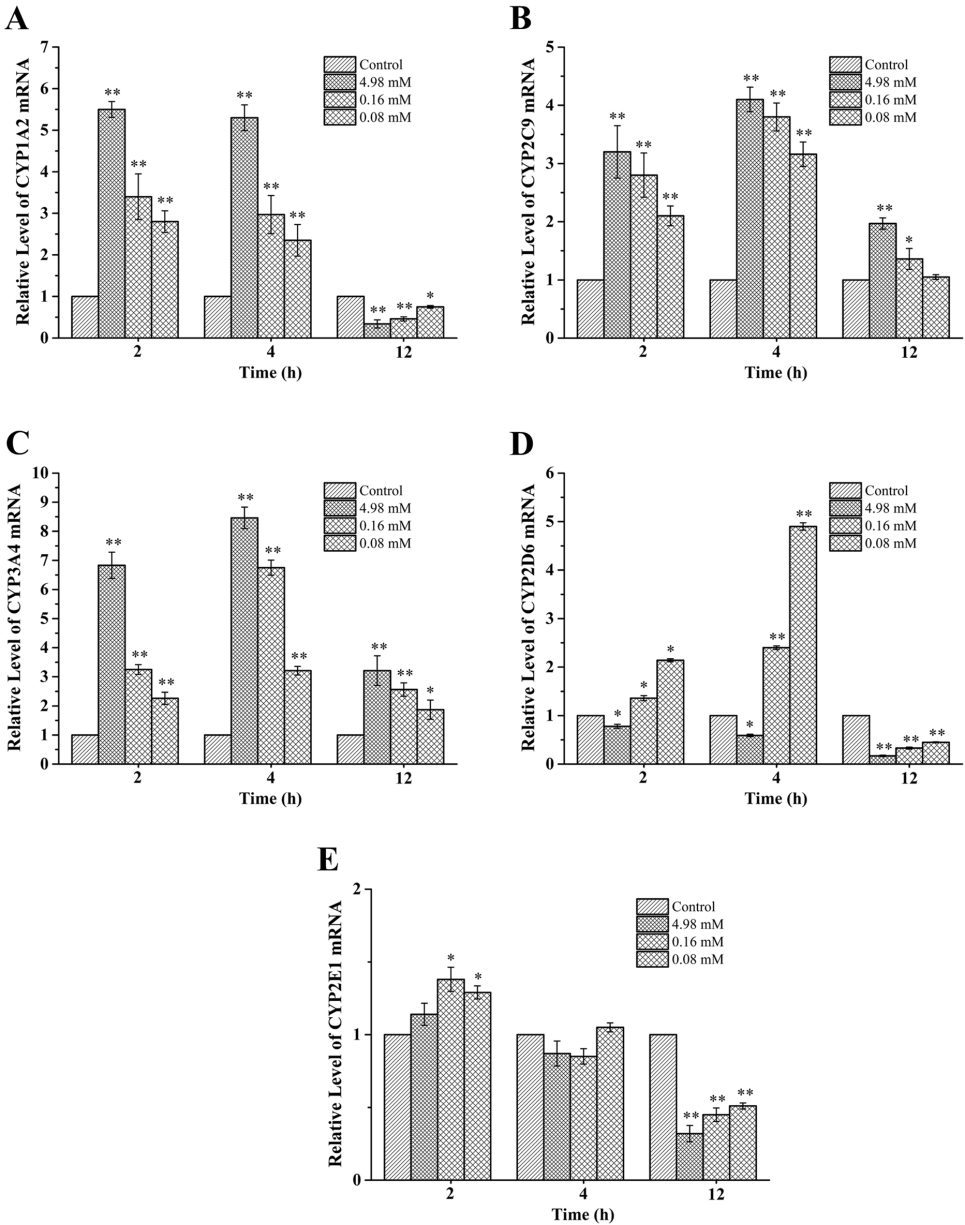
In vitro effect of DPTM on the mRNA expression in Hep G2 cells. CYP 450 enzymes include CYP1A2 (**A**), CYP2C9 (**B**), CYP3A4 (**C**), CYP2D6 (**D**) and CYP2E1 (**E**). Data are the mean ± SD (n = 3). *P < 0.05 and **P < 0.01 versus the control group.

To further investigate the inhibitions of DPTM against CYP isoforms, the expressions of the regulated genes of CYP1A2, CYP2D6, CYP2E1, CYP2C9 and CYP3A4 and their transcriptional regulators (AhR, PXR and PPAR) were evaluated using the Hep G2 cell model. Compared to the control, treatment for 2 and 4 h with 4.98, 0.16 and 0.08 mM DPTM resulted in significantly increase in CYP1A2, CYP2C9 and CYP3A4 mRNA expressions (Fig. 4A–C) and showed dose-dependent effect. When DPTM treated for 12 h with 4.98 and 0.16 mM, CYP2C9 and CYP3A4 mRNA expressions were significantly higher than that of control (Fig. 4B,C), but CYP1A2, CYP2D6 and CYP2E1 mRNA expressions were significantly lowered than that of control when treated for 12 h (Fig. 4A,D,E). However, no significantly difference of CYP2E1 mRNA level between three doses of DPTM and control was found after treatment for 4 h (Fig. 4E).

**Figure 5:**
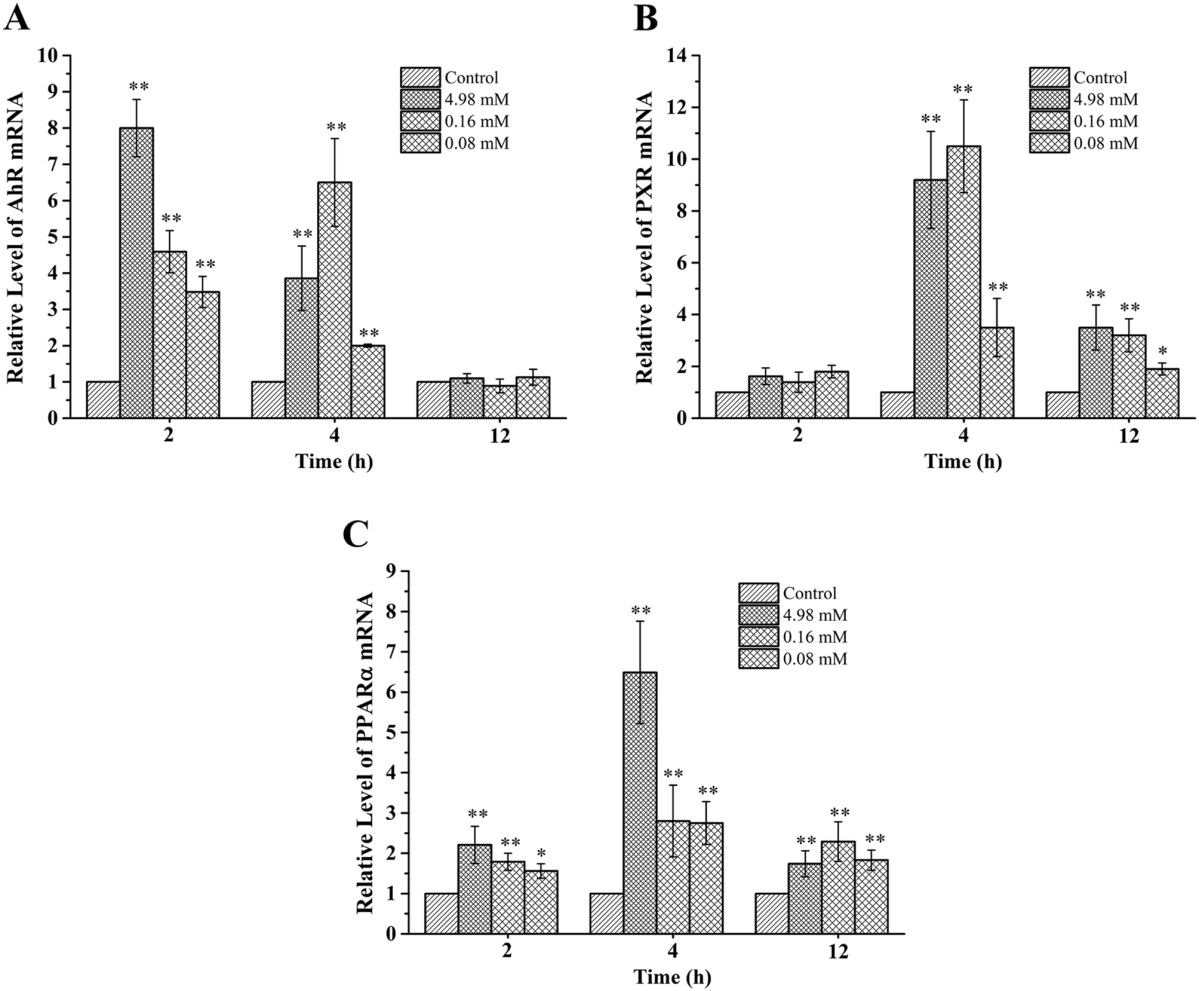
DPTM increase the mRNA expression of AhR (**A**), PXR (**B**) and PPARα (**C**) in Hep G2 cells. Data are the mean ± SD (n = 3). *P < 0.05 and **P < 0.01 versus the control group.

Compared to the control, treatment with 4.98, 0.16 and 0.08 mM DPTM for 2 and 4 h significantly increased the AhR mRNA content, while there was no effect when treated for 12 h (Fig. 5A). Three doses of DPTM significantly increased the PXR mRNA expression when treated for 4 and 12 h (Fig. 5B). Compared to the control, three doses of DPTM could significantly increase PPARα mRNA expression when treated for 2, 4 and 12 h (Fig. 5C).

### Ames test

The genotoxicity of DPTM was conducted by bacterial reverse mutation assay against five mutant *S*. *typhimurium* strains with or without S9 metabolic activation . The results showed revertant colonies were observed in all positive groups and were much more in double than that induced by five concentrations of DPTM and DMSO (Table 2). However, there was no significantly different between the number of revertant colonies induced by DPTM and the negative control (DMSO), which indicated that DPTM should not be considered as a mutagen^20^. The number of colonies induced by DPTM also showed no dose-dependent effect based on this test.

**Table 2:** Ames test results of DPTM using *S. typhimurium* strains TA97, TA98, TA100, TA102 and TA1535.

Substance	Dose (μmol/plate )	TA97		TA98		TA100		TA102		TA1535	
		+ S9	− S9	+ S9	− S9	+ S9	− S9	+ S9	− S9	+ S9	− S9
DPTM	3.98	182 ± 12	169 ± 14	30 ± 7	28 ± 11	142 ± 22	173 ± 15	231 ± 19	268 ± 12	16 ± 3	22 ± 4
	1.99	184 ± 13	174 ± 17	29 ± 8	32 ± 8	145 ± 14	170 ± 12	245 ± 20	264 ± 15	13 ± 5	22 ± 3
	1.00	173 ± 13	171 ± 8	33 ± 9	27 ± 8	151 ± 18	165 ± 14	223 ± 12	260 ± 17	14 ± 3	26 ± 1
	0.50	168 ± 17	165 ± 14	27 ± 11	30 ± 10	140 ± 10	162 ± 13	227 ± 9	241 ± 10	19 ± 4	21 ± 5
	0.25	175 ± 17	70 ± 11	29 ± 6	33 ± 6	165 ± 14	171 ± 15	235 ± 20	256 ± 20	15 ± 2	22 ± 3
Negative control		181 ± 20	159 ± 12	28 ± 6	31 ± 6	160 ± 11	169 ± 11	231 ± 26	250 ± 25	19 ± 2	22 ± 4
Positive control		2,986 ± 209	803 ± 37	1,464 ± 348	164 ± 16	1,071 ± 133	984 ± 59	2008 ± 157	690 ± 33	347 ± 21	774 ± 20

The numbers indicate the means and standards deviation values of CFU in triplicate assay systems.

+ S9: with metabolic activation; − S9: without metabolic activation.

Negative control: DMSO (0.2 mL/plate).

Positive control: 2-aminoanthracene for all strains with S9; 4-nitro-o-phenylenediamine for TA97 without S9; daunorubicin for TA98 without S9 , sodium azide for TA 100 and TA 1535 without S9, mitomycin for TA102 without S9.

## Discussion

Drug-drug interaction potential and genotoxic activity of novel drugs are important properties for their development and essential to guide appropriate use in patients. This study evaluated the in vitro CYP enzymes inhibition activities and the genotoxicity of DPTM, a novel pleuromutilin derivative with a substituted pyrimidine moiety.

In the present studies, the purity of DPTM (98.72%) was below 99.00%, which was not enough pure to examine its inhibitory effects and genotoxic property. According to the synthetic scheme16 and HPLC chromatography of DPTM (see Supplementary Fig. S1), sodium *p*-toluenesulfonate and *p*-toluenesulfonic acid, the two know genotoxic impurities, showed strongly water soluble and could be completely removed when the reaction mixture was washed with water after the chemical reactions. Pleuromutilin, 4,6-diamino-2-mercaptopyrimidine and 14-O-(p-Toluene-sulfonyloxyacetyl)-mutilin, the main process impurities, could be neglected toxicity properties because they were usually used as pharmaceutical raw materials. Therefore, in spite of the low purity of DPTM, the biased results of its CYP enzymes inhibition activity and the genotoxicity could be negligible.

Before the bioactivities studies on the cellular level, it is necessary to evaluate the cytotoxic potential of a drug. The cell viability against DPTM was 79% with the concentration of 4.98 mM, indicating that DPTM showed lower cell toxicity and could be used for the further studies on the Hep G2 cell model with concentration not more than 4.98 mM.

CYP enzymes are very important for the metabolism of a wide range of endogenous compounds, as well as xenobiotics including drugs, environmental pollutants and dietary products^21–23^. Some chemicals affect the disposition of conventional pharmaceuticals through the inhibition of CYP enzymes^24,25^. Therefore, we used the cocktail method to determine the substrate content of CYP1A2, CYP2D1/6, CYP2E1, CYP2C9/11 and CYP3A/4 in RLMs and HLMs for evaluating the inhibition activity of DPTM at different concentrations. Our study showed that DPTM displayed a moderate inhibitory effect on CYP3A4, similar to retapamulin^8,26^, but apparently lower than that of azamulin with IC_50_ value of 0.24–0.03 µM, and 14-O-((5-Amino-benzimidazole-2-yl) thioacetyl) mutilin (with IC_50_ value of 1.69 µM)^12,27^. However, the selective inhibition for CYP3A4 may be a primary function of the pleuromutilin portion of the molecule^12,28^. Furthermore, the side chain structure of pleuromutilin derivative has some influence on CYP3A4 inhibition profiles^29^.

For further evaluating the inhibition activities of DPTM on the CYP isoforms, we investigated CYP enzymes and their main transcription factors (AhR, PXR and PPAR) mRNA expressions using human HepG2 cell-line. The main transcription factors AhR, PXR and PPAR are responsible for the induction of one to four CYP families (AhR for CYP1A2, PXR for CYP2C9 and CYP3A4, and PPAR for CYP2E1)^30,31^. The present study showed DPTM could increase the mRNA expressions of CYP isoforms. However, the significantly decrease of the mRNA expressions of CYP1A2, CYP 2D6 and CYP 2E1 when treated with DPTM for 12 h (Fig. 4) may be related to the rapid metabolic property of DPTM with half-life (t_1/2_) of 1.7–1.9 h in rats after intravenous administration^32^. We speculated that HepG2 cells metabolize DPTM rapidly within a short time. After treatment for 12 h, the majority of prototype drug was changed to one or more new compounds that probably inhibited the metabolic function of the cells. However, this mechanism was obtained on the basis of speculation and should be further confirmed.

The mRNA expressions of transcriptional regulators were also increased when HepG2 cells were treated by DPTM (Fig. 5). Interestingly, this compound displayed the similar effect on the CYP enzymes mRNA expressions.

Ames test is a rapid, effective and short-term bacterial reverse mutation assay which is commonly employed as an initial screening for genotoxic activity of chemical substances *in vitro*^33,34^. The present study was carried out using five *S. typhimurium* strains TA97, TA98, TA100, TA102 and TA1535 with and without S9 metabolic activation. We did not observe any treatment-related mutagenic activity in the histidine auxotrophy of the *S. typhimurium* strains up to 3.98 μmol/plate, indicating that under this study conditions, they do not produce reverse mutations in five *S. typhimurium* strains either in the presence or absence of metabolic activation system. Furthermore, no linear relationship of number of colonies induced by five doses of DPTM was observed with or without S9.

In conclusion, DPTM displayed moderate inhibitory potentials on CYP3A/4 and weak against CYP1A2, CYP2D1/6, CYP2E1 and CYP2C11/9 in RLMs and HLMs using cocktail method. Furthermore, this compound significantly increased the mRNA expressions of CYP isoforms and their transcriptional regulators when HepG2 cells were treated for a certain period of time. The Ames test showed that DPTM did not exhibit mutagenic response under the present experimental conditions. These results provide useful data and information for further developing DPTM to a potential anti-bacterial clinical candidate.

## Material and methods

### Materials

DPTM was synthesized in our laboratory using the standard technique^16^. The purity of this compound was checked by HPLC and quantitative NMR analyses with 98.72% (see Supplementary S1), and its structure was confirmed by IR, NMR and HR-MS spectrometry. Acetonitrile, methyl alcohol and formic acid were of liquid chromatography-mass spectrometry (LC–MS) grade and purchased from Fisher Scientific (New Jersey, USA). Ultrapure water was purchased from A.S. Watson Group (Guangzhou) Ltd. (Guangzhou, China) and used throughout the study. Tolbutamide (CAS No. 64-77-7), metoprolol tartrate (CAS No. 56392-17-7), phenacetin (CAS No. 62-44-2), chlorzoxazone (CAS No. 95-25-0), dapsone (CAS No. 80-08-0), ketoconazole (CAS No. 65277-42-1), fluvoxamine (CAS No. 54739-18-3), glucose-6-phosphate (CAS No. 5996-17-8), glucose-6-phosphate dehydrogenase (CAS No. 9001-40-5) and nicotinamide adenine dinucleotide phosphate (NADP, CAS No. 53-59-8) were purchased from Sigma-Aldrich (St. Louis, USA). 2-Aminoanthracene (CAS No. 613-13-8) was purchased from Wako Pure Chemical Industries (Chuo-ku, Japan). 4-Nitro-o-phenylenediamine (CAS No. 99-56-9), daunorubicin (CAS No. 23214-92-8), sodium azide (CAS No. 26628-22-8) and mitomycin (CAS No. 99-56-9) were purchased from Acros Organics (Trenton, USA). Trizol reagent was purchased from Beijing Suo Laibao Technology Co., Ltd. (Beijing, China). Diethylpyrocarbonate (DEPC) treated water was purchased from Leagene Biotechnology (Beijing, China). All the other chemicals were of analytical grade and obtained from commercial sources.

Hep G2 cells were purchased from Beina Biotechnology Co., Ltd. (Beijing, China). RPMI-1640 culture media and SYBR Green Real-time PCR Master Mix were purchased from Thermo Fisher Scientific (Carlsbad, USA). Foetal bovine serum was purchased from Bovogen Biologicals Pty Ltd (East Keilor, Australia). Male Pooled SD Rat Liver S9, male SD rat liver microsomes and pooled human liver microsomes were all procured from Research Institute for Liver Diseases (Shanghai) Co., Ltd (Shanghai, China). PrimeScript RT reagent Kit was purchased from Takara Biotech Co., Ltd. (Kusatsu, Japan). *Salmonella typhimurium* (*S. typhimurium*) strains TA97, TA98, TA100, TA102 and TA1535 were purchased from Molecular Toxicology, Inc. (MolTox), USA (Blue Ridge Mountains, USA)^35^.

### Methods

#### Culturing of Hep G2 and cytotoxicity assay

Intrinsic toxicity of DPTM was conducted as described in the literature^36–38^ with some modifications. Briefly, 100 μL/mL of RPMI-1640 medium containing 10% foetal bovine serum and Hep G2 cells was seeded in each well of a flat-bottom 96-well plate. After proliferating to logarithmic growth phase at 37 °C under 5% CO_2_, cells were seeded to a new 96-well plate with a density of 4 × 10^6^ cells/hole and cultured for 24 h at the same condition. The media were replaced with 100 μL of serial dilutions of DPTM (10 mg DPTM was dissolved in 1 mL 1% DMSO to a solution and then diluted two-fold with distilled water to provide 19.91, 9.96, 4.98, 2.49, 1.25, 0.62, 0.31, 0.16 and 0.08 mM serial dilutions). The blank group only used 1% DMSO to replace the media. The obtained mixture was incubated for 24 h. After that, all media in cells were removed and 20 μL of MTT reagent was added for an additional 4 h. Then, supernatant fraction was removed and 100 μL dimethyl sulfoxide (DMSO) was added, followed by incubation at 37 °C for 15 min. The absorbance of each well was determined by a spectrophotometer at dual wavelengths of 570 and 279 nm for the background. The viability percentage was calculated by the following formula: cell viability % = (OD_sample_ − OD_blank_/OD_control_ − OD_blank_) × 100. A sigmoid-shaped curve was fitted to the data, and the IC_50_ was calculated by fitting the Hill equation to the data with nonlinear regression (least-squares best fit modelling) using GraphPad Prism 4 (GraphPad Software Inc., San Diego, USA). The measurements were repeated at least three times.

#### In vitro assay of CYP activity in RLMs and HLMs

We used the cocktail method to study the inhibition activities of DPTM against CYP enzymes in RLMs and HLMs with slight modification^39^. DPTM (250 μmol) was dissolved in 1.25 mL methanol to a solution and then diluted with methanol and water to 2, 10, 20, 100, 200, 400, 1,000 and 2000 μM, respectively. The obtained serial dilutions (10 μL) were added to the incubation mixtures (200 μL total volume), containing 100 μL RLMs or HLMs (final concentration was 5 μg/μL, respectively), 10 μL glucose-6-phosphate (final concentration was 1 mM), 10 μL glucose-6-phosphate dehydrogenase (final concentration was 0.1 U/L), 20 μL potassium phosphate buffer (final concentration was 0.01 M) and 20 μL MgCl_2_ (final concentration was 2 mM), to give the final concentrations were 0.1, 0.5, 1, 5 10, 20, 50, and 100 μM, respectively. For the groups that in the absence of DPTM, 10 μL methanol (1%) replaced the DPTM and was added to the incubation mixtures. The substrates (phenacetin for CYP1A2, tolbutamide for CYP2C9/11, metoprolol for CYP2D1/6, chlorzoxazone for CYP2E1 and dapsone for CYP3A/4) were dissolved methanol and added at final concentrations of 10 μM (55 μM for dapsone). The final concentration of methanol in all the incubation mixtures, including the blank group, was less than 0.2% (v/v). After being pre-incubated at 37 °C for 5 min, the reaction was initiated by adding 20 μL NADPH with the final concentration of 1 mM. The reaction was quenched by adding 400 μL of acetonitrile after being incubated for 15 min. The mixtures were centrifuged at 10,000 rpm for 10 min, and an aliquot of supernatant was transferred to an auto-injector vial for analyzing the five substrates using a previously validated HPLC method^40^. The determined metabolic substrates were used for calculating the relative enzyme activity (E_rel_) by the following formula: E_rel_ (%) = Ci(n)/Ci(0) × 100%, in which Ci(n) was the determined metabolic substrates in the presence of varying concentrations of DPTM and Ci(0) was the determined metabolic substrates in the absence of DPTM. According to the obtained relative enzyme activities and inhibitor concentrations, the IC_50_ values were obtained as the same method as the cytotoxicity of DPTM. Various concentrations of fluvoxamine (final concentrations were 0.02, 0.05, 0.1, 0.5, 1, 5 10 and 20 μM) and ketoconazole (final concentrations were 0.01, 0.02, 0.05, 0.1, 0.5, 1, 5 and 10 μM) which replaced DPTM for CYP1A2 and CYP3A4 were performed as the same procedure. The same procedure was repeated three times.

#### Specific mRNA expression in Hep G2 cells

The logarithmic growth phase Hep G2 cells were seeded into 12-well plastic plates at a density of 4 × 10^7^ cells/well. After incubation at 37 °C under 5% CO_2_ for 24 h, the media were then replaced with 1 mL of serial dilutions of the DPTM (4.98, 0.16 and 0.08 mM) in fresh media. When cells were incubated for 2, 4 and 12 h at 37 °C under 5% CO_2_, the RNA was isolated using the Trizol reagent according to the manufacturer’s protocol. Total RNA (5 μL) was transcribed into cDNA by using the PrimeScript RT reagent Kit (Perfect Real Time). The obtained cDNA (5 μL) was added for 45 cycles of PCR amplification to detect the mRNA expression (the primers and sequences used herein were given in Table 3) using the ABI Villa TM7Dx real-time PCR system (Gaithersburg, USA). Glyceraldehyde-3-phosphate dehydrogenase (GAPDH) was used as reference gene. All reactions were performed in a final volume of 20 μL (10 μL of 2 × SYBR Green Real-time PCR Master Mix, 2 μL of cDNA and 2 μL of the primers and 6 μL DEPC water) and executed in triplicate.

**Table 3:** Primer and sequence for real-time PCR.

Gene Name	Sequence (5′ → 3′)
GAPDH	FP: AAGAAGGTGGTGAAGCAGGC
	RP: GCGTCAAAGGTGGAGGAGTG
CYP2B6	FP: CGGATTCAGGAGGAGGCT
	RP: GCAGATGATGTTGGCGGT
CYP2C8	FP: AATGGAAAGAGATGGAAGGAGAT
	RP: AGCACAGCCCAGGATGAAAG
CYP2C9	FP: GGGACAGAGACGACAAGCA
	RP: TGTAGCACAGAAGTCAGGGAAA
CYP2C19	FP: TCCCAAGGGCACAACCATA
	RP: CCTCTCCCACACAAATCCG
CYP1B1	FP: CACCTCTGTCTTGGGCTACC
	RP: ACTGAAAAAATCATCACTCTGCT
CYP3A4	FP: CGAAGATACACAAAAGCACCGA
	RP: TTCATAGCCAGCAAAAATAAAGATAA
CYP3A5	FP: ATTCCTTACCCCAGTTTTTGA
	RP: GGTGCTTTTGTTTGTCGTTG
CYP2D6	FP: GCAAGGTCCTACGCTTCCAA
	RP: CTCAGTCAGGTCTCGGGGG
CYP1A1	FP: GCCTCTGTCATCTTCTGTCTGG
	RP: CATACTGCTGGCTCATCCTTG
AHR	FP: CTTTATTGTGCCGAGTCCCA
	RP: CTCTGTTCCTTCCTCATCTGTTAGT
CAR	FP: CTCCTGCTGTGCTTCGTGCT
	RP: GCAGTTTCCCCTTTGGCTTT
PXR	FP: CGGAAGAAAAGTGAACGGACA
	RP: TCGGAGAAGGTAGTGTCAAAGGT
PPARα	FP: GTCATCACGGACACGCTTT
	RP: CCCCGCAGATTCTACATTC

*FP* forward primer, *PR* reverse primer,* AhR * aryl hydrocarbon receptor,* PXR* pregnane X receptor,* PPARα* peroxisome proliferator-activated receptor α.

#### Ames test

*S. typhimurium* strains TA97, TA98, TA100, TA102 and TA1535 were stored and maintained as provided in the standard protocol^41^. Each of the strains was checked for their genetic integrity prior to the experiment, and tested for biotin dependence, histidine dependence, biotin and histidine dependence, *rfa* marker (crystal violet) and presence of the plasmid pKM101 (ampicillin resistance) or pAQ1 (tetracycline resistance). Different strains were used to identify different types of mutations.

The test was carried out at five different concentrations of DPTM. DPTM (39.84 μmol) was weighed accurately and dissolved in 2 mL DMSO. Then 1 mL solution was diluted with distilled water by two fold. The obtained serial dilutions (1 mL) were then incorporated into 9 mL melted top agar to dilute to the desired concentrations (1.99, 1.00, 0.50, 0.25 and 0.13 μmol/mL). The negative control used in this experiment was 1 mL DMSO which was directly incorporated into 9 mL melted top agar. 2-aminoanthracene was used as positive control for all bacterial strains in the presence of S9 with the concentration of 13.3% (v/v). Four different positive control chemicals were used for each tested strain without S9, including 4-nitro-o-phenylenediamine (10 μg/plate) for TA97, daunorubicin (2 μg/plate) for TA98, sodium azide (5 μg/plate) for TA 100 and TA 1535, and mitomycin (0.5 μg/plate) for TA102.

The Ames test of DPTM was conducted as described in the literatures^42,43^ with some modifications. Briefly, 1 mL of strain was inoculated into 5 mL of nutrient broth, followed by incubating overnight at 37 °C. The mixture comprised respective strains, drug, negative and positive controls with the S9 for testing in the presence of metabolic activation system. For testing without S9, 1 × phosphate buffer saline (PBS, pH 7.4) replaced the S9 mix. The obtained mixtures were added into the tube, mixed well and incubated at 37 °C for 20 min. Then, the top agar was melted and 2 mL of this melted top agar was added to each tube and gently mixed. The mixture was poured onto the surface of glucose minimal (GM) agar plate (the final concentrations of DPTM were 3.98, 1.99, 1.00, 0.50 and 0.25 μmol/plate and the final concentration of DMSO was 0.2 mL/plate, respectively). The plate was then swirled to distribute the overlay agar to all surfaces of GM agar, followed by invertion and incubation at 37 °C for 48 h when the top agar solidified. The resultant colonies were counted manually considering the high density of bacterial colony. The results were expressed as the number of revertant colonies per plate. The experiments were performed in triplicate.

#### Data analysis

The data were presented as mean ± standard deviation (SD). Statistical analyses were performed by One-way ANOVA followed by the Line Segment Detector (LSD) test using SPSS 17.0 software. Differences were considered significant at *p* < 0.05.

## Supplementary information

Supplementary information.
