# Supplementary material for: Cytochrome P450 inhibition potential and initial genotoxic evaluation of 14-O-[(4,6-diaminopyrimidine-2-yl)thioacetyl] mutilin
Source: Sci Rep. 2020 Aug 10;10:13474. doi: 10.1038/s41598-020-70400-8 (PMC7417534; doi:10.1038/s41598-020-70400-8)
Supplement: Supplementary file 1 — Supplementary information. [file 41598_2020_70400_MOESM1_ESM.pdf]

Cytochrome P450 inhibition potential and initial genotoxic evaluation of 14-O-[(4,6-Diaminopyrimidine-2-yl)thioacetyl] motilin

Yunxing Fu[1,2], Yunpeng Yi[1], Yuan Fan[1], Ruofeng Shang[1]*

[1]*Key Laboratory of New Animal Drug Project of Gansu Province; Key Laboratory of Veterinary Pharmaceutical Development, Ministry of Agriculture, P. R. China; Lanzhou Institute of Husbandry and Pharmaceutical Sciences of CAAS, 730050 Lanzhou, China*

[2]*Henan University of Animal Husbandry and Economy, Zhengzhou 450046, China*

Supplementary Data

Table of Contents

1. The method for determining DPTM purity……………………………………………………………………1

2. Supplementary Fig. S1. NMR spectra of DPTM and IS…………………………………………………………2

3. Supplementary Fig. S2. HPLC chromatogram of DPTM ……………………………………………………………3

1. Supplementary Note-The method for determining DPTM purity

Because DPTM was a new structural chemical, and no reference standard of DPTM was obtained to determine its purity with conventional method, we used the HPLC (area normalization) and quantitative NMR analyses to determine DPTM purity.

The HPLC method

The analysis was carried out on an Agilent1290 HPLC (California, USA) system equipped with a solvent degasser, a quaternary pump with controller, a manual injector and a diode array detector (DAD-2998). The ZORBAX SB-C18 column (250 × 4.6 × 5 μm) was used for the separation with the column temperature maintained at 30 ℃. The injection volume was 10 μL and the total run time was 22.5 min. The isocratic elution with a mobile phase of acetonitrile-0.02 mol/L KH_2PO_4 buffer solution (50:50, v/v) pumped at a flow rate of 1.0 mL/min throughout the HPLC process. The mobile phase was filtered through a 0.22 μm filter and degassed ultrasonically for 15 min before use. The DPTM was detected at the absorption wavelength of 205 nm.

Stock solution of DPTM was prepared at concentration of 100 μg/mL in acetonitrile and further diluted into 10.00 μg/mL for the preparation of working solution. The obtained working solution was injected in HPLC system (repeated in triplicate) for determining DPTM purity using area normalization method. The mean of three chromatographic peak areas was used as DPTM purity.

The quantitative NMR analyses

We used Bruker-400 MHz spectrometer (Zürich State, Switzerland) to record the ^{1}H NMR spectra of DPTM and 1,4-dinitrobenzene (99.9%) used as an internal standard (IS) in $CDCl_3$ for five times. The obtained data were processed by MestReNova software. The NMR peaks of DPTM in δ = 6.42 (C_{19}-H) and δ = 5.68 (C_{14}-H), as well as the NMR peak of IS in δ = 8.35 (four Hs in benzene ring), were chosen as quantitative peaks (see Supplementary Fig. S1) to calculate the purity of DPTM with the formula:

$$P_x = \frac{I_x}{I_{std}} \times \frac{N_{std}}{N_x} \times \frac{M_x}{M_{std}} \times \frac{m_{std}}{m} \times P_{std}$$

Note: M_x and M_{std} were molecular weights of DPTM and IS, respectively; m and m_{std} were the DPTM and IS weights used for analyses, respectively; I_x and I_{std} were the selected NMR peak areas of DPTM and IS, respectively; N_{std} and N_x were the proton number of selected groups in DPTM and IS, respectively; P_{std} was the purity of IS.

The purity of DPTM

The mean value of the obtained data of HPLC and quantitative NMR was calculated as the final purity of DPTM.

2. Supplementary Fig. S1. NMR spectra of DPTM and IS

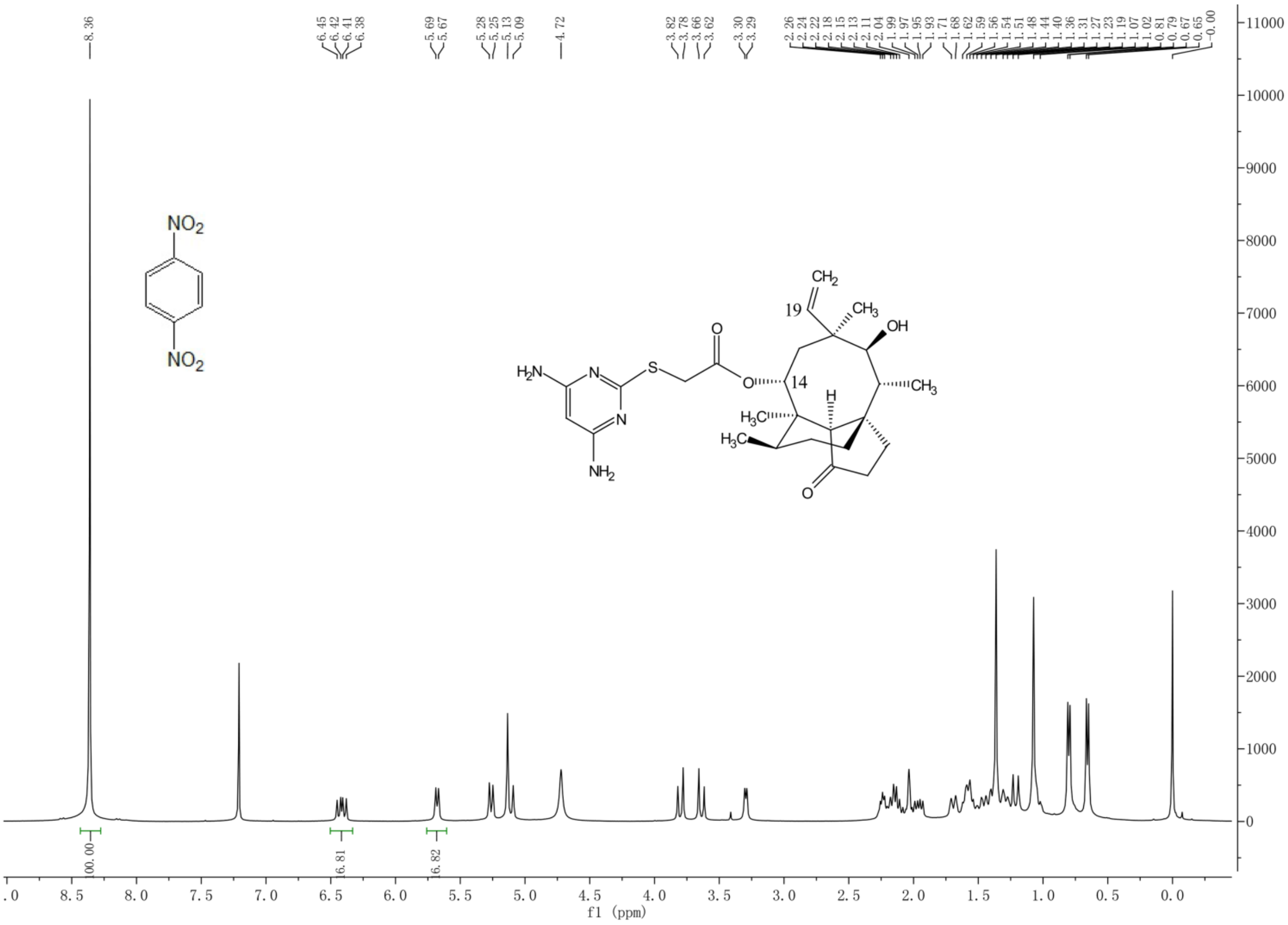

3. Supplementary Fig. S2. HPLC chromatogram of DPTM

数据文件: C:\CHEM32\1\DATA\DPTM含量测试 2018-04-17 16-52-53\1DB-0201.D
样品名称: DPTM含量测试

===

操作者 : 系统 序列行 : 2
仪器 : 1290LC 位置 : P1-D-02
进样日期 : 2018/4/17 17:19:21 进样次数 : 1
进样量 : 1.000 µl
采集方法 : C:\CHEM32\1\DATA\DPTM 含量测试 2018-04-17 16-52-53\DPTM质量标准.M
最后修改 : 2018/4/17 17:40:38 : 系统
(调用后修改)

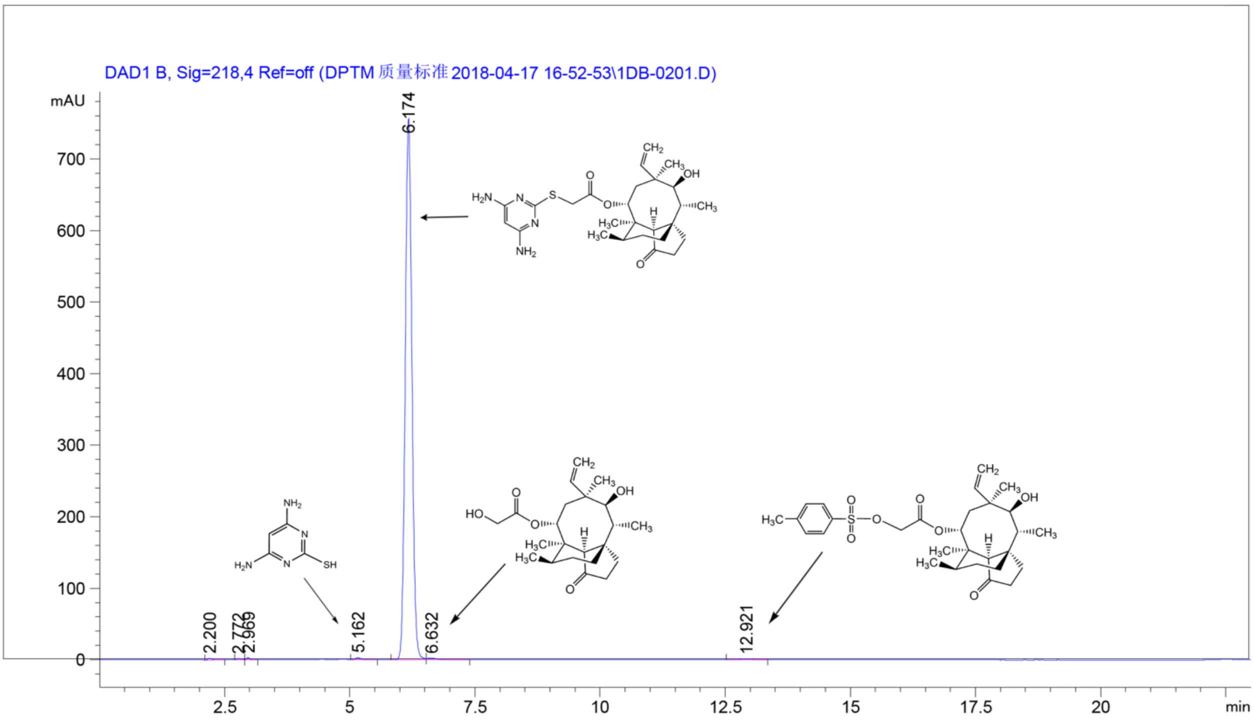

===

面积百分比报告

===

排序 : 信号
乘积因子 : 1.0000
稀释因子 : 1.0000
内标使用乘积因子和稀释因子
稀释因子

信号 1: DAD1 B, Sig=218,4 Ref=off

峰 #	保留时间 [min]	类型	峰宽 [min]	峰面积 [mAU*s]	峰高 [mAU]	峰面积 %
1	2.200	BB	0.1078	16.78267	2.28293	0.2360
2	2.772	BV	0.0994	11.44646	1.68098	0.1610
3	2.969	VB	0.0646	13.16263	3.03712	0.1851
4	5.162	VB	0.1375	24.40032	2.64734	0.3431
5	6.174	BV	0.1438	6991.66602	756.89429	98.3123
6	6.632	VB	0.1984	33.41513	2.34288	0.4699
7	12.921	BB	0.2694	20.81680	1.14875	0.2927
总量 :				7111.69001	770.03428	

===

*** 报告结束 ***
